# Current status of PET imaging in Huntington’s disease

**DOI:** 10.1007/s00259-016-3324-6

**Published:** 2016-02-22

**Authors:** Gennaro Pagano, Flavia Niccolini, Marios Politis

**Affiliations:** Neurodegeneration Imaging Group, Department of Basic & Clinical Neuroscience, Maurice Wohl Clinical Neuroscience Institute, Institute of Psychiatry, Psychology & Neuroscience (IoPPN), King’s College London, 125 Coldharbour Lane, Camberwell, London SE5 9NU UK

**Keywords:** Huntington’s disease, Premanifest Huntington’s disease gene carriers, Neurodegeneration imaging, Positron emission tomography

## Abstract

**Purpose:**

To review the developments of recent decades and the current status of PET molecular imaging in Huntington’s disease (HD).

**Methods:**

A systematic review of PET studies in HD was performed. The MEDLINE, Web of Science, Cochrane and Scopus databases were searched for articles in all languages published up to 19 August 2015 using the major medical subject heading “Huntington Disease” combined with text and key words “Huntington Disease”, “Neuroimaging” and “PET”. Only peer-reviewed, primary research studies in HD patients and premanifest HD carriers, and studies in which clinical features were described in association with PET neuroimaging results, were included in this review. Reviews, case reports and nonhuman studies were excluded.

**Results:**

A total of 54 PET studies were identified and analysed in this review. Brain metabolism ([^18^F]FDG and [^15^O]H_2_O), presynaptic ([^18^F]fluorodopa, [^11^C]β-CIT and [^11^C]DTBZ) and postsynaptic ([^11^C]SCH22390, [^11^C]FLB457 and [^11^C]raclopride) dopaminergic function, phosphodiesterases ([^18^F]JNJ42259152, [^18^F]MNI-659 and [^11^C]IMA107), and adenosine ([^18^F]CPFPX), cannabinoid ([^18^F]MK-9470), opioid ([^11^C]diprenorphine) and GABA ([^11^C]flumazenil) receptors were evaluated as potential biomarkers for monitoring disease progression and for assessing the development and efficacy of novel disease-modifying drugs in premanifest HD carriers and HD patients. PET studies evaluating brain restoration and neuroprotection were also identified and described in detail.

**Conclusion:**

Brain metabolism, postsynaptic dopaminergic function and phosphodiesterase 10A levels were proven to be powerful in assessing disease progression. However, no single technique may be currently considered an optimal biomarker and an integrative multimodal imaging approach combining different techniques should be developed for monitoring potential neuroprotective and preventive treatment in HD.

## Introduction

Huntington’s disease (HD) is an autosomal dominant monogenic neurodegenerative disease with a prevalence of 0.4 – 5.7 per 100,000 worldwide [1]. HD is caused by an expanded CAG trinucleotide repeat sequence in the huntingtin gene on chromosome 4, which leads to the formation of intranuclear inclusions of mutated huntingtin in the brain, resulting in loss of GABAergic medium spiny neurons (MSNs) in the striatum and in cortical areas [2]. HD is clinically characterized by motor symptoms (chorea and parkinsonism), cognitive symptoms (slowed mentation, attention, mental flexibility, planning and emotion recognition) and psychiatric symptoms (depression, apathy, impulsivity, irritability, disinhibition and psychosis), with a progressive course and a typical onset in adult middle age (40 – 55 years) [3]. The age at onset is inversely correlated with the size of the CAG repeat expansion [4]. This allows us to predict the motor onset [5] and classify the disease in stages such as premanifest HD (before motor onset) and manifest HD (after motor onset) [6] providing a tremendous opportunity to investigate subclinical and pathological changes in asymptomatic HD gene carriers. This offers a therapeutic window for potential preventive treatments aiming to delay the clinical onset of HD.

The mechanisms underlying the progressive neurodegeneration in HD are still unclear and, currently, there is no single proven biomarker that allows us to monitor disease progression and assess the efficacy of novel disease-modifying drugs. The lack of biomarkers may be related to the fact that HD pathology causes only minor brain alterations in early stages [7].

Molecular imaging techniques are able to identify subtle alterations at the nanomolecular level and this is a prerequisite to understanding minimal changes in brain activity [8, 9]. PET molecular probes bind a target, such as a receptor, a transporter or an enzyme, with high specificity and power of resolution [10]. PET molecular imaging has revolutionized the ability to gain insights into human brain biology and beyond this to understand the physiology and pathophysiology of neurological diseases [11]. PET radiotracers have provided invaluable insights into the natural history of HD and have been used to measure brain metabolism, dopaminergic function, neuroinflammation, phosphodiesterases and other targets in HD [12]. They have contributed to the identification of disease characteristics at different stages mainly in cross-sectional studies but also in some longitudinal studies.

This review describes the developments during recent decades and current applications of PET molecular imaging techniques in HD.

## Methods

### Search strategy

The MEDLINE, Web of Science, Cochrane CENTRAL and Scopus databases were searched for articles in all languages published up to 19 August 2015. Studies were identified and evaluated by two of the authors (G.P. and F.N.) using the major medical subject heading “Huntington Disease” combined with text and key words “Huntington Disease”, “Neuroimaging” and “PET”. Additional eligible studies were identified by screening the reference lists of the studies found.

### Inclusion criteria

Studies were excluded if the title and/or abstract was not appropriate for the aim of the review. The full text of eligible studies and of studies whose relevance was uncertain were obtained. Selected studies were eligible if they met the following criteria: (1) peer-reviewed, primary research studies, (2) studies including HD patients, (3) studies including a description of the clinical features of the HD patients in association with neuroimaging results, and (4) studies including PET neuroimaging. Reviews, case reports and nonhuman studies were excluded.

## Results

Of 944 articles identified by the initial search, 86 were retrieved for more detailed evaluation, and 54 PET studies were finally identified and analysed in this review. The results of 32 of the main studies are summarized in Table 1.

**Table 1:** Main PET studies in Huntington’s disease

Year	Reference	Study population	PET tracer	Molecular target	Main findings
Brain metabolism					
1997	[15]	7 HD, 7 HCs	[^15^O]H_2_O	Regional cerebral blood flow	Deficit in the task-related activation in the striatum and its frontal motor area projections
1990	[19]	23 HD, 21 HCs	[^18^F]FDG	Glucose uptake	Decrease in caudate and cortical metabolism. Correlation with cognitive dysfunction
1996	[17]	8 HD, 10 pre-HD, previous HCs	[^18^F]FDG, [^11^C]raclopride	Glucose uptake, D_2_ receptors	Lower loss of glucose uptake than of D_2_ receptor levels (2.3 % vs. 6.3 % per year)
2006	[23]	47 HD, 24 pre-HD, 30 HCs	[^18^F]FDG	Glucose uptake	Decrease in caudate and cortical metabolism in HD patients and in pre-HD patients. Hypometabolism in pre-HD patients preceded volume loss
2012	[24]	46 pre-HD	[^18^F]FDG	Glucose uptake	Lower caudate metabolism in patients with pre-HD converted to HD than in patients with pre-HD not converted (5-years follow-up)
2013	[28]	12 pre-HD, 12 HCs	[^18^F]FDG, [^11^C]raclopride	Glucose uptake, D_2_ receptors	Progressive alterations of striatothalamic and cortical metabolic activity network in pre-HD patients
Dopaminergic function					
1994	[36]	5 HD, 1 pre-HD, 5 HCs	[^11^C]SCH 23390	D_1_ receptors	Decrease (75 %) in striatal D_1_ receptors density in HD patients
1995	[39]	10 HD patients, 9 HCs	[^11^C]SCH 23390, [^11^C]raclopride	D_1_ receptors, D_2_ receptors	Decrease in striatal D_1_ and D_2_ receptors in HD patients. Akinetic-rigid patients had greater D_1_ and D_2_ receptor loss in the striatum than choreic HD patients
1997	[33]	5 HD, 5 HCs	[^11^C]SCH 23390, [^11^C]raclopride, [^11^C]β-CIT	D_1_ receptors, D_2_ receptors, DAT	Decrease in striatal D_1_ and D_2_ receptors and striatal DAT binding. Correlation between D_2_ receptor loss and duration of symptoms
1998	[45]	8 HD, 10 pre-HD	[^11^C]Raclopride	D_2_ receptors	Decrease in D_2_ receptors in the striatum of pre-HD patients. Correlation between D_2_ receptor loss and CAG repeat length
1998	[43]	17 pre-HD	[^11^C]SCH 23390, [^11^C]raclopride	D_1_ receptors, D_2_ receptors	Decrease in striatal D_1_ and D_2_ receptors in pre-HD patients. Correlation between D_1_ and D_2_ receptor loss and cognitive dysfunction
1999	[37]	4 HD, 9 pre-HD, 7 HCs	[^11^C]SCH 23390, [^11^C]raclopride	D_1_ receptors, D_2_ receptors	Decrease in striatal D_1_ and D_2_ receptors in pre-HD patients. Correlation between D_1_ and D_2_ receptor loss and UHDRS
2000	[34]	19 HD, 64 HCs	[^11^C]DTBZ	VMAT2	Decrease in VMAT2 binding in caudate (30 %) and putamen (60 %)
2005	[38]	27 pre-HD	[^11^C]Raclopride, [^18^F]FDG	D_2_ receptors, glucose uptake	Decrease (50 %) in D_2_ receptors in the striatum of pre-HD patients. Correlation between D_2_ receptor loss and increases in the product of age and CAG repeat length
2006	[40]	12 HD, previous HCs	[^11^C]Raclopride	D_2_ receptors	Decrease in D_2_ receptors in the amygdala, and temporal and frontal cortex in HD patients
2008	[44]	9 HD, 10 pre-HD, 10 HCs	[^11^C]Raclopride, [^11^C]PK11195	D_2_ receptors, TSPO	Decrease in D_2_ receptors in the hypothalamus of HD and pre-HD patients. Correlation between D_2_ receptor loss and increased activation of microglia
2010	[42]^a^	16 HD, 11 pre-HD, previous HCs	[^11^C]Raclopride	D_2_ receptors	Decrease in D_2_ receptors in the cortex of HD and pre-HD patients. Correlation between D_2_ receptor cortical levels and cognitive dysfunction
2011	[35]	9 HD, 9 HCs	[^11^C]FLB457	D_2_ receptors	Decrease in D_2_ receptors in the striatum but not in extrastriatal areas. Correlation between striatum D_2_ receptor loss and motor symptoms
Activation of microglia					
2006	[51]^a^	11 HD, 10 HCs	[^11^C]PK11195, [^11^C]raclopride	TSPO, D_2_ receptors	Increase in activation of microglia in striatal and cortical areas in HD patients. Correlation between increased TSPO and loss of D_2_ receptors in the striatum and with motor dysfunction (UHDRS)
2007	[52]^a^	11 pre-HD, 10 HCs	[^11^C]PK11195, [^11^C]raclopride	TSPO, D_2_ receptors	Increase in activation of microglia in striatal and cortical areas in pre-HD patients. Correlation between increased TSPO and loss of D_2_ receptors in the striatum
2011	[53]^a^	8 pre-HD, 8 HCs	[^11^C]PK11195, [^11^C]raclopride	TSPO, D_2_ receptors	Increase in activation of microglia in striatal and cortical areas in pre-HD patients. Correlation between increased TSPO in associative striatum and cognitive dysfunction
2015	[54]^a^	12 pre-HD, 12 HCs	[^11^C]PK11195	TSPO	Increase in activation of microglia in cortical regions, basal ganglia and thalamic region in pre-HD patients. Correlation between increased TSPO and plasma levels of IL-1β, IL-6, IL-8 and TNF-α. First in vivo evidence of an association between peripheral and central immune responses in pre-HD patients
PDE10A					
2014	[59]	5 HD, 11 HCs	[^18^F]JNJ42259152	PDE10A	Decreases in PDE10A of 70.7 % and 62.6 % in the caudate and putamen, respectively. No correlations between PDE10A and clinical scales
2014	[60]	8 HD, 3 pre-HD, 9 HCs	[^18^F]MNI-659	PDE10A	Decreases in PDE10A of 50 % in the striatum of manifest HD patients compared with HCs. Pre-HD patients had values intermediate between HD patients and HCs. Striatal correlations between PDE10A and severity of disease measured by the clinical scale (UHDRS, motor subscale), the molecular marker (BOP), and regional atrophy
2015	[61]	12 pre-HD, 12 HCs	[^11^C]IMA107	PDE10A	Decreases in PDE10A of 30 % in the striatum and 25 % in the pallidus, and increase of 35 % in motor thalamic nuclei in pre-HD patients
Adenosine and cannabinoid receptors					
2014	[64]	8 HD, 6 pre-HD-B^b^, 7 pre-HD-A^c^, 36 HCs	[^18^F]CPFPX	A_1_ receptors	Cerebral A_1_ receptor levels in pre-HD-A patients were higher than in HCs (by up to 31 % in the thalamus). Successive reduction in A_1_ receptor binding in pre-HD-B patients compared with HCs and binding in manifest HD patients lower than in HCs (decrease to 25 % in the caudate and amygdala). Strong correlation between A_1_ receptor binding and years since onset
2010	[73]	20 HD, 14 HCs	[^18^F]MK-9470, [^18^F]FDG	CB_1_ receptors, glucose uptake	Decrease in CB_1_ receptors in the cerebrum and cerebellum (grey matter) and brainstem
Opioid and GABA receptors					
1993	[71]	6 HD, 6 HCs	[^11^C]Flumazenil, [^18^F]FDG	GABA receptors, glucose uptake	Decrease in GABA receptors in the caudate and decrease in glucose uptake in the caudate putamen and thalamus
2000	[72]	10 HD, 13 pre-HD	[^11^C]Flumazenil, [^18^F]FDG, [^11^C]raclopride	GABA receptors, glucose uptake, D_2_ receptors	Decrease in GABA receptors in the caudate and decrease in glucose uptake and D_2_ receptors in the caudate, putamen and thalamus in HD patients. Reduction in GABA receptors was significantly less in manifest HD patients than in pre-HD patients with normal D_2_ receptor levels, but not in comparison with pre-HD patients with reduced D_2_ receptor levels
1997	[77]	2 HD, 9 HCs	[^11^C]Diprenorphine	Opioid receptors	Decrease in opioid receptors in the caudate and putamen

*A*_*1*_ adenosine type 1, *CB*_*1*_ cannabinoid type 1, *D*_*1*_ dopaminergic type 1, *D*_*2*_ dopaminergic type 2, *HC* healthy controls, *PDE10A* phosphodiesterase 10A, *UHDRS* unified Huntington’s disease rating scale

^a^Cortical changes were not present in all the patients included in this study

^b^pre-HD-B: near predicted symptom onset

^c^pre-HD-A: far from predicted symptom onset

### Brain metabolism

[^15^O]H_2_O and [^18^F]FDG have been used as markers of cerebral blood flow and cerebral glucose metabolism providing an index of neuronal integrity and the functional state of neurons [13]. Changes in motor-related cortical activation have been evaluated using [^15^O]H_2_O PET [14, 15]. In manifest HD, impaired activation of the striatum and its frontal motor projection areas during motor tasks, such as paced joystick movements or sequential finger-to-thumb opposition, has been found. There was a parallel increased activity in the parietal areas [14] and insular areas [15]. These findings suggest that the loss of MSNs in the striatum leads to impairment of the basal ganglia-thalamocortical motor output and may induce a compensatory recruitment of additional accessory motor pathways [14, 15]. Moreover, different patterns of brain activation have been shown in HD patients during word generation task [16]. During the word generation task, HD patients show decreased cerebral blood flow in the anterior cingulate and the inferior frontal gyri (related to lexical selection) and a compensatory activation of the left supramarginal gyrus and the right inferior frontal gyrus, suggesting the presence of compensatory language strategies in HD [16].

[^18^F]FDG studies in manifest HD patients have shown glucose hypometabolism in the striatum and the cortex [17–19]. Striatal hypometabolism was associated with motor dysfunction [20], and cortical hypometabolism with cognitive dysfunction [19, 21]. Striatal and cortical hypometabolism has also been found to precede neuronal loss in premanifest HD gene carriers [22–24]. However, it has been suggested that a possible hypermetabolism precedes the decrease in these areas [25]. Moreover, [^18^F]FDG hypometabolism was not localized in a specific area, and an approach using a network of regions with altered metabolism has been used to identify spatial covariance patterns in premanifest HD [25–28]. Following this approach, relative bilateral increases in thalamic, occipital and cerebellar glucose metabolism associated with bilateral decreases in striatal metabolism has been identified as a characteristic pattern of HD, which discriminated well between HD patients and healthy controls [26]. This characteristic pattern has shown good reliability as a marker of disease progression although there was a tendency to be quite stable after an initial impairment (nonlinear trend), thus limiting its clinical utility [26]. Reduction in [^18^F]FDG uptake in the striatum, and frontal, parietal and temporal cortex indicates an impairment in intracellular processes, such as calcium handling, transcription regulation and mitochondrial energy production, while an increase in [^18^F]FDG uptake in the thalamus, and occipital and cerebellar cortex might indicate an increased glycolytic response due to mitochondrial dysfunction [25] or due to the lack of inhibitory activity exerted by the damaged basal ganglia on the thalamocortical system [26].

### Dopaminergic function

Pathognomonic chorea and the other motor features (parkinsonism and dystonia) of HD are related to impairment in dopaminergic function [29]. The neurodegenerative process mainly affects the striatal MSNs expressing dopaminergic type 1 (D_1_) and type 2 (D_2_) receptors [30]. This leads to severe involvement of the postsynaptic dopaminergic system with relatively spared presynaptic dopaminergic nerve terminals. However, presynaptic dopaminergic function in HD has been investigated only in small and older studies, with discordant results. In manifest HD patients, caudate [^18^F]fluorodopa uptake (targeting dopa decarboxylase) has been found to be reduced [31] or normal [32], [^11^C]β-CIT levels (targeting striatal dopamine transporter) were 50 % reduced [33] and [^11^C]DTBZ levels (targeting type-2 vesicular monoamine transporter) showed a gradient from a rostral increase to a caudal decrease in the striatum [34]. However, in these PET studies no correction for partial volume effects was performed and their results may have been affected by regional volume loss, and thus it is unclear whether they measured degeneration of neurons or pure presynaptic dopaminergic terminal dysfunction. Presynaptic dopaminergic function has not been investigated in premanifest HD patients, nor has the association with CAG repeat expansion or other HD-related clinical features.

Postsynaptic dopaminergic dysfunction is a key characteristic of HD. This has been investigated using [^11^C]SCH22390 as marker of D_1_ receptors density and using [^11^C]raclopride and [^11^C]FLB457 [35] as markers of D_2_ receptor density. The densities of D_1_ and D_2_ receptors were significantly reduced in the striatum, ranging from 40 % [33] to 75 % [36] in HD patients and from 25 % [37] to 50 % [38] in premanifest HD gene carriers. This loss was found to be higher in akinetic-rigid HD patients than in choreic manifest HD patients [39]. Reductions in striatal D_1_ and D_2_ receptors were also correlated with worse motor dysfunction, as assessed using the unified Huntington’s disease rating scale (UHDRS) [37, 40]. In extrastriatal areas, a reduction in D_1_ receptors has been found in the temporal cortex and was associated with cognitive dysfunction in manifest HD patients [33, 41]. In a study using [^11^C]raclopride including 16 manifest HD patients and 11 premanifest HD gene carriers [42], the cortical D_2_ receptor levels were reduced in both manifest HD patients and premanifest HD gene carriers and were associated with the degree of cognitive dysfunction (attention and executive functions). In line with these results, another study using [^11^C]raclopride [40] showed a reduction in D_2_ receptor levels in the amygdala, and frontal and temporal cortex of 12 manifest HD patients. Levels of both D_1_ and D_2_ receptors (but mainly D_2_ receptors) were strongly correlated with cognitive performance in 17 premanifest HD gene carriers [43]. Hypothalamic D_2_ receptor levels were also significantly reduced in presymptomatic and premanifest HD gene carriers [44]. The early involvement of the hypothalamus might explain the prodromal nonmotor symptoms and could be a potential early biomarker in HD. The number of CAG repeat lengths has been associated with a reduction in striatal D_2_ receptor levels in premanifest HD patients [38, 45], suggesting that mutant huntingtin plays a role in the expression of dopamine receptors. These findings support the primary involvement of sensorimotor striatum dysfunction in the motor component of the disease [40] and of associative striatum and temporal cortex dysfunction in the cognitive component of the disease [42], with D_2_ receptor loss as the common biological substrate.

### Activation of microglia

Activation of microglia is considered one of the mechanisms underlying neurodegeneration [46]. However, it is not clear if it represents a compensatory mechanism for neuronal loss or a trigger of damage [47]. Microglia release cytokines when stimulated by abnormal proteins, such as mutant huntingtin. These cytokines are proinflammatory and further stimulate microglia, resulting in a self-propagating cascade, which may lead to neuronal dysfunction and death [48]. This process is not confined to the brain, but peripheral plasma cytokines are also increased in premanifest and manifest HD gene carriers [47]. Activation of microglia can be measured using 18-kDa translocator protein (TSPO) ligands, such as [^11^C]PK11195, [^11^C]GE180, [^11^C]PBR28, and others [49]. So far only [^11^C]PK11195 has been used to measure TSPO in HD gene carriers. [^11^C]PK11195 is a first-generation TSPO PET ligand, whereas the others were developed recently and represent second-generation TSPO PET [50]. Increased activation of striatal and cortical microglia has been found in manifest HD gene carriers [51] and in premanifest HD gene carriers and was associated with loss of striatal D_2_ receptors ([^11^C]raclopride binding) [52]. In manifest HD patients, striatal TSPO levels are also associated with motor dysfunction (UHDRS) [52]. In a more recent study, increased TSPO binding has been observed specifically in the associative striatum of premanifest HD patients and correlated with cognitive dysfunction [53]. We have also recently demonstrated that plasma levels of IL-1β, IL-6, IL-8 and TNF-α are correlated with increased [^11^C]PK11195 binding in the somatosensory cortex of premanifest HD patients [54]. These findings demonstrate for the first time in vivo a link between peripheral and central immune dysfunction and support the role of immune dysfunction in the pathogenesis of HD. In terms of pathology, it is reasonable to assume that the more dorsal part of the striatum (related to motor and cognitive function) is affected earliest in HD, whereas the ventral part of the striatum (related to psychiatric function) is affected later, possibly after the onset of motor symptoms [53].

### Phosphodiesterases

Phosphodiesterases are a family of intracellular enzymes hydrolysing cyclic nucleotides (cAMP and cGMP) [55]. Phosphodiesterase 10A (PDE10A) is mainly expressed in striatal MSNs where it regulates the cAMP/PKA/DARPP-32 signalling cascade, and thus plays a key role in the regulation of striatal output and in promoting neuronal survival [55, 56]. PDE10A has received increasing attention after the observation that its pharmacological inhibition in an animal model of HD significantly improves HD symptoms and pathology [57]. Post-mortem studies have confirmed that PDE10A is severely reduced in manifest HD patients [58].

**Fig. 1:**
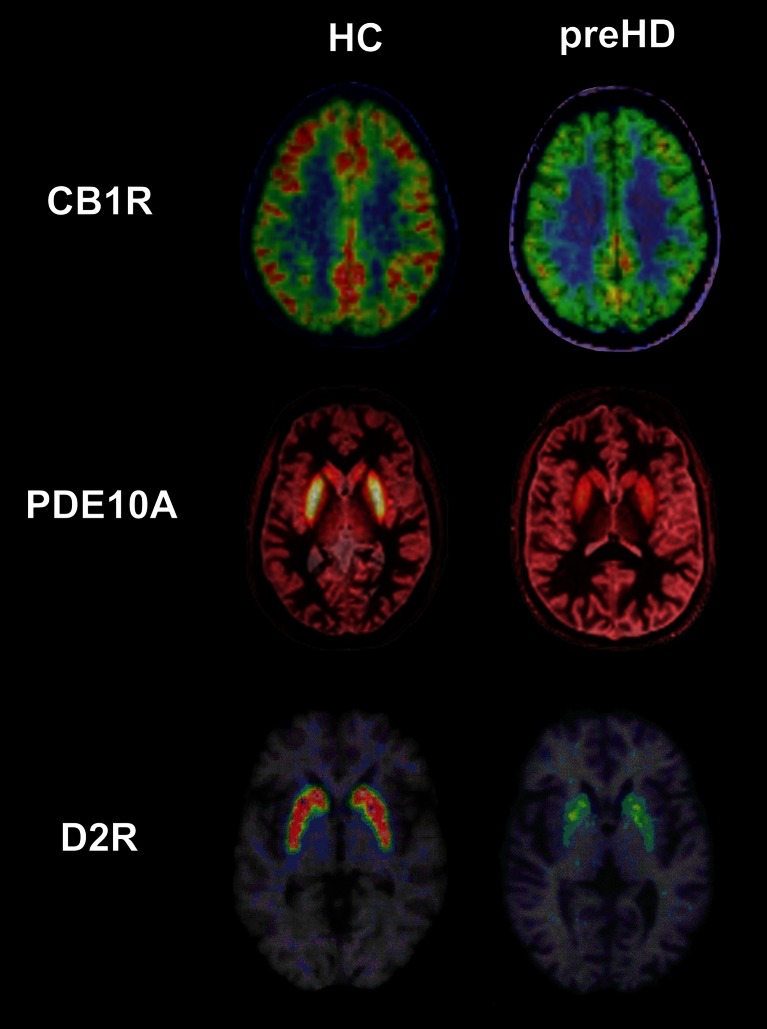
Example PET images in healthy controls (HC) and premanifest Huntington’s disease gene carriers (preHD). Axial [^11^C]MePPeP, [^11^C]IMA107, [^11^C]raclopride images in stereotaxic space overlaid onto the T1-weighted MNI template show decreased cortical CB_1_ receptor, striatal PDE10A and striatal D2 receptor binding in the preHD subjects compared to the healthy controls . Images from our data

[^18^F]JNJ42259152, [^18^F]MNI-659 and [^11^C]IMA107 have been used to quantify of PDE10A expression in vivo in HD patients [59–61]. In five manifest HD patients, Ahmad et al. [59] found 70.7 % and 62.6 % [^18^F]JNJ42259152 binding reductions in the caudate and putamen, respectively (Fig. 1). These authors found no correlations between [^18^F]JNJ42259152 levels and clinical scales. Russell et al. [60], found 47.6. % decreases in striatal and pallidal [^18^F]MNI-659 binding in eight patients with early manifest HD. Lower striatal [^18^F]MNI-659 binding was associated with worse UHDRS motor scores, disease burden of pathology and regional atrophy. In three premanifest HD gene carriers, who were a mean of 12 years from predicted onset, striatal PDE10A expression was also found to be decreased although to a lesser degree compared to the group of manifest HD patients [60].

 Using [^11^C]IMA107 PET, our group has recently investigated PDE10A expression in 12 HD gene carriers with early premanifest asymptomatic HD who were a mean of 25 years before the predicted onset of motor symptoms [61]. We found 25 – 33 % reductions in striatal and pallidal [^11^C]IMA107 binding, and 35 % increases in the motor thalamic nuclei. We then combined PET and diffusor tensor imaging MRI data to perform a connectivity-based striatal parcellation analysis of PDE10A expression according to (a) cortex–striatum connectivity profiles and (b) striatal connections with the globus pallidus externus and substantia nigra/globus pallidus internus, which reflect the major parts of the *indirect* and *direct* pathways, respectively. We found that PDE10A decreases were confined to the sensorimotor striatum and to the striatonigral and striatopallidal projecting segments. Moreover, the altered balance of PDE10A expression between the motor thalamic nuclei and the striatopallidal internal projecting segments of the striatum was strongly associated with the predicted risk of symptomatic conversion. Our findings are a major breakthrough in the field of HD in showing the earliest biochemical abnormality identified in HD to date, and showing bidirectional alteration of PDE10A expression within the neuropathological salient networks in HD gene carriers up to 43 years before the development of overt clinical symptoms, which are crucial to prognosis. These findings provide in vivo evidence that PDE10A plays a role in the pathophysiology of HD, although longitudinal studies are needed to confirm the relationship between PDE10A and clinical symptomatology.

### Adenosine and cannabinoid receptors

The identification of novel targets is a primary objective of current PET research in HD. Type 1 adenosine (A_1_) and type 1 cannabinoid (CB_1_) receptors represent only a few among many potential novel targets that have recently become investigable with PET imaging. In animal studies, adenosine was increased by about 50 % in a mouse model of HD [62], and A_1_ receptor levels in the hypothalamus were found to be lower in HD rats carrying 51 CAG repeats [63]. [^18^F]CPFPX has been used as a marker of A_1_ receptors in HD [64]. Matusch et al. [64] found a 25 % reduction in [^18^F]CPFPX binding in the caudate of eight manifest HD patients (Fig. 1). They studied premanifest HD patients, dividing them into premanifest gene carriers far from predicted symptom onset (preHD-A; *n* = 7) and near to predicted symptom onset (preHD-B; *n* = 6). Interestingly, in preHD-A patients, [^18^F]CPFPX binding in the thalamus was 31 % higher than in healthy controls, while in preHD-B patients, thalamic [^18^F]CPFPX binding was similar to the levels in healthy controls. There was a strong correlation between A_1_ receptor binding and years to onset [65]. These findings suggest that A_1_ receptors switch from supranormal to subnormal levels during phenoconversion of HD. This differential regulation may play a role in the pathophysiology of altered energy metabolism.

CB_1_ receptors are mainly expressed in GABA-ergic striatal MSNs [65] (also expressing D_1_ and D_2_ receptors) and are a key modulator of inhibitor neurotransmission [66]. In post-mortem studies in manifest HD patients, a reduction in CB_1_ receptor levels was demonstrated in the striatum [67] and GABA receptors were decreased in the caudate and putamen and increased in the globus pallidus [68] and substantia nigra [69]. This indicates that output GABAergic projections from the striatum are severely affected in HD patients [70]. This hypothesis has been confirmed in vivo in PET studies conducted to evaluate GABA/benzodiazepine receptors [71, 72] and CB_1_ receptors [73].

[^18^F]MK-9470 has been used as a marker of CB_1_ receptors in PET studies of the cannabinoid system in HD [73]. Van Laere et al. [73] found reduced CB_1_ receptor binding in the grey matter of the cerebrum and cerebellum, and in the brainstem of 20 patients with manifest HD (Fig. 1). They used a simplified semiquantification model and their findings need to be confirmed in a full quantification model. The widespread CB_1_ receptor changes observed in HD are in line with the suppressive effect of mutant huntingtin on CB_1_ transcription [74]. This finding strongly suggests that cannabinoid dysfunction is heavily involved in HD even during the early presymptomatic stages of the disease.

### Opioid and GABA receptors

Old targets, such as opioid and GABA, might be reintroduced in combination with novel ones. Post-mortem studies have shown that the opioid system is also affected in HD [75, 76]. A loss of encephalin of striatal projection neurons to the external globus pallidus has been reported in premanifest HD [75] and a reduction in the proencephalin/prodynorphin ratio in the caudate, putamen, and internal and external globus pallidus has been found in manifest HD patients [76]. [^11^C]Diprenorphine has been used as a marker of opioid receptor in HD [77]. Significant reductions of 31 % and 26 % in [^11^C]diprenorphine binding in the caudate and putamen, respectively, were demonstrated in five manifest HD patients [77]. These reductions were lower than striatal dopaminergic degeneration (about 60 % in manifest HD [39]), suggesting that opioid receptor sites are less vulnerable to disease processes than dopamine sites, possibly because of preservation of presynaptic opioid sites.

[^11^C]Flumazenil has been used as marker of GABA/benzodiazepine receptors [71, 72]. Holthoff et al. [71] found a 17 % reduction in [^11^C]flumazenil binding in the caudate of six manifest HD patients, with normal putamen and thalamic binding. They also found a reduction in [^18^F]FDG binding of 47 %, 41 %, 18 % in the caudate, putamen and thalamus, respectively. In ten manifest HD patients, Künig et al. [72] found the same results, showing reduced [^11^C]flumazenil binding in the caudate (with normal values in the putamen) and reduced [^18^F]FDG binding in the caudate and putamen. The metabolic impairment of the putamen and thalamus, without detectable loss of benzodiazepine receptor density, confirms that the reduction in [^18^F]FDG uptake happens before the changes in [^11^C]flumazenil binding. Künig et al. also compared [^11^C]flumazenil and [^18^F]FDG binding with [^11^C]raclopride binding in 13 premanifest HD patients. [^11^C]Raclopride binding in the caudate and putamen was reduced in all manifest HD patients and in eight premanifest patients. As previously reported, a reduction in D_2_ receptor levels indicates neuronal cell loss. Thus, they divided premanifest gene carriers into two groups: those with and those without a decrease in [^11^C]raclopride. [^11^C]Flumazenil binding in the caudate was significantly lower in manifest HD patients compared with premanifest patients without a decrease in [^11^C]raclopride, but not compared with those with a decrease in [^11^C]raclopride who displayed [^11^C]flumazenil values between those of the control subjects and the manifest HD patients. These findings indicate that the loss in pallidocaudate GABA projections was marked only in manifest HD patients. [^11^C]Flumazenil binding in the putamen was not different among healthy controls, and premanifest and manifest HD gene carriers. The authors suggest that the normal [^11^C]flumazenil binding in the putamen of manifest HD patients might be a compensatory mechanism in the pallidal projection areas of striatal GABAergic MSNs. However, these PET studies were not corrected for partial volume effects and the results may have been affected by regional volume loss.

No studies investigating the cannabinoid system in premanifest HD gene carriers have been published. Interestingly, CB_1_ receptors and PDE10A transcription is modulated by huntingtin and, in HD animal models expressing a mutant huntingtin, CB_1_ receptors and PDE10A were strongly reduced [78]. This reduction was present not only in the symptomatic stage but also in the asymptomatic stage, even before decreases in glucose uptake. Thus, these proteins may not only be excellent biomarkers of HD progression but may also represent novel therapeutic targets for HD.

### Progression monitoring

Several longitudinal studies have been conducted in HD patients to identity biomarkers, monitor disease progression and evaluate novel treatments. Brain metabolism and dopaminergic function have been evaluated as potential PET biomarkers. In a longitudinal study, Feigin et al. [26] observed a progressive decline in [^18^F]FDG uptake across three time-points over 4 years. A recent [^18^F]FDG PET study has shown that premanifest HD gene carriers who become symptomatic after 5 years following a PET scan have a mean glucose uptake in the caudate significantly lower than those who do not convert, and this difference is independent of CAG repeats [24]. These findings suggest that brain metabolic changes may be predictors of HD symptomatic conversion. Decreased cortical metabolism in the early stage of HD is considered indicative of rapid progression [79]. Indeed, cortical metabolism in the frontotemporal and parietal cortices is significantly lower in patients with early HD with faster progression of the disease as measured using the Independence Scale of the UHDRS [79].

A distinct spatial covariance pattern has been identified by Tang et al. [28] as associated with disease progression using longitudinal metabolic imaging data in premanifest HD patients. This pattern showed a progressive impairment in striatothalamic and cortical metabolic activity over 7 years and was not influenced by symptomatic conversion. Moreover, premanifest HD patients at higher risk of symptomatic conversion were those with higher metabolic network activity at baseline [28]. Thus, metabolic network may be a sensitive biomarker for disease progression during presymptomatic stages.

In a combined [^18^F]FDG and [^11^C]raclopride longitudinal study, premanifest HD gene carriers showed an annual loss of 2.3 % in striatal glucose metabolism and 6.3 % in D_2_ receptor binding [17]. These findings suggest that glucose metabolism is a less sensitive marker of disease progression than [^11^C]raclopride [17]. In another longitudinal study, the decline in D_2_ receptor levels was constant (around 5 % per year) [40], although no correlation between changes in UHDRS motor scores and reductions in striatal binding were observed [40]. This may be explained by the sigmoid model, in which the motor symptoms reach a plateau (threshold of caudate degeneration/hypometabolism) and further degeneration is not associated with worsening of symptoms. In premanifest HD patients, longitudinal [^11^C]raclopride studies have shown rates of decline from 4 % [37] up to 6.3 % [17]. In a 40-month longitudinal study [37], nine premanifest HD patients and four manifest HD patients showed a mean annual D_1_ receptor binding reductions of 2 % and 5 %, respectively, and D_2_ receptor binding reductions of 4 % and 3 %, respectively [37]. Among them, premanifest HD patients with annual losses ≥5 % demonstrated faster disease progression. Thus, striatal D_1_ and D_2_ receptor binding may be used to identify asymptomatic HD subjects with a higher risk of conversion [37]. However, in another study [80] reduction in putaminal dopamine D_2_ receptor binding was weakly correlated with the probability of symptomatic conversion within 5 years, as calculated using a model based on age and CAG repeats, and the rate of change of putaminal D_2_ receptor binding was not increased around the time of HD symptom onset [80]. Striatal degeneration might proceed in a non-linear fashion in HD. A correlation between CAG repeat length and the estimated percentage loss of striatal D_2_ receptor binding were found in a cross-sectional study among premanifest and manifest HD subjects [45]. However, while CAG repeat length influenced the rate of disease progression, the slopes of the correlation for premanifest and manifest HD population were significantly different, suggesting that the rate of disease progression is faster during the earlier asymptomatic stages of the disease [45]. Therefore, striatal D_2_ receptor measures should be used mainly in patients during premanifest HD stages, when they are more sensitive.

Recently, novel biomarkers have been used to monitor disease progression. Russell et al. [81] found reduced striatal [^18^F]MNI-659 uptake of about 50 % at baseline in eight manifest HD patients compared with seven healthy controls. At 1 year, [^18^F]MNI-659 uptake had declined in the basal ganglia in all eight manifest HD patients with mean annualized rates of reduction in of 16.6 % in the caudate, 6.9 % in the putamen, and 5.8 % in the globus pallidus. A decline in clinical status as assessed using UHDRS was also observed in this cohort. To date, three subjects had completed imaging assessment at 2 years and they showed a mean annualized reduction in [^18^F]MNI-659 uptake at similar rate to that found at 1 year (caudate 15.5 %, putamen 7.2 %, and globus pallidus 9.4 %). Despite these promising results, head-to-head studies need to be performed comparing [^18^F]MNI-659 with [^18^F]FDG and [^11^C]raclopride to finally determine which functional PET markers are most sensitive for monitoring HD progression.

Differences found between HD patients and healthy subjects in the uptake of PET tracers in various brain regions are summarized in Table 2.

**Table 2:** PET tracer uptake changes in different brain area of HD patients compared with HC subjects

Brain target	Tracer	Caudate	Putamen	Thalamus	Globus pallidus	Frontal	Parietal	Temporal	Occipital
Brain metabolism	[^18^F]FDG	−	−	+	NE	−	−	−	+
D_1_ receptors	[^11^C]SCH 23390	−	−	NE	NE	=	NE	−^b^	NE
D_2_ receptors	[^11^C]Raclopride	−	−	NE	NE	−^b^	NE	−^b^	=
Activation of microglia	[^11^C]PK11195	+	+	=	+	+	+	=	=
PDE10A	[^18^F]JNJ42259152	−	−	+^a^	−	NE	NE	NE	NE
	[^18^F]MNI-659								
	[^11^C]IMA107								
A_1_ adenosine receptors	[^18^F]CPFPX	−	−	−	NE	−	−	−	=
CB_1_ receptors	[^18^F]MK-9470	−	−	NE	NE	NE	NE	NE	NE
OPIOID receptors	[^11^C]Diprenorphine	−	−	+	NE	+	NE	−	=
GABA receptors	[^11^C]Flumazenil	−	=	=	=	=	=	=	=

− reduced, + increased, = normal, *NE* not evaluated

^a^Evaluated only in preHD

^b^Not in all the patients

Unfortunately, current biomarkers did not show consistent results in terms of changes over time, thus limiting their utility as markers of disease progression. A novel model (sigmoid acceleration–deceleration) recently applied in Alzheimer’s disease [82] might also be useful in HD. This model has been suggested to identify biomarkers with a nonlinear reduction over time and assumes an initial acceleration of volume loss up to an inflection point following which deceleration of atrophy occurs [82]. The pathophysiological changes in HD extend far beyond the striatum, cortical changes occur even at the earliest stages of the disease. Using structural MRI, cortical thinning has been found in early manifest HD patients [83] and cortical degeneration of premotor and supplementary motor areas has been found to be associated with bradykinesia [84]. Functional MRI studies have shown reorganization of cortical circuitry at the early stage of HD [85]. Using an “interference” task that activates multiple brain regions including the anterior cingulate, inferior parietal, inferior temporal, sensorimotor, premotor and inferior frontal cortices, different patterns of brain activation have been found in premanifest HD gene carriers further from disease onset (increased activation) compared with premanifest HD gene carriers closer to disease onset (reduced activation) [85].

### Brain restoration

Brain metabolism and dopamine receptors levels have been used to assess the efficacy of restorative therapy in HD patients. A multicentre open-label pilot study was designed to evaluate the safety and efficacy of bilateral fetal striatal transplantation [86]. Transplantation was performed in five HD patients who were followed clinically and with PET over 3 – 10 years [87, 88]. No significant differences were found over time using the UHDRS between patients who had and had not received the transplant and striatal D_1_ and D_2_ receptor binding indicated that there was no obvious surviving striatal graft tissue [87, 88]. A 2-year follow-up study with [^18^F]FDG in five HD patients who underwent bilateral striatal transplantation [89, 90] was performed to assess the restoration of striatocortical function [90]. Three patients showed an increase in striatal/cortical glucose metabolic rate, associated with clinical improvement or stabilization suggesting restoration of striatocortical function. However, a 2-year follow-up multicentre study failed to show significant this change in [^18^F]FDG uptake [87]. Therefore, whether bilateral striatal transplantation may restore striatocortical connections remains a matter of debate [91, 92].

### Neuroprotective drugs

Brain metabolism has been used to assess the efficacy of potential neuroprotective drugs in HD. A randomized-controlled trial using riluzole was performed in 23 HD patients (11 treated with riluzole and 12 with placebo) [93]. After a mean follow-up of 24 months placebo-treated patients showed a significantly greater proportional volume loss of grey matter and decrease in metabolic [^18^F]FDG uptake than patients treated with riluzole in all cortical areas. The decreased rate of metabolic [^18^F]FDG uptake correlated with worsening clinical scores in placebo-treated patients [93]. A small open-label trial using pridopidine was performed in eight HD patients [94] and increased metabolic activity in several brain regions including the precuneus and mediodorsal thalamic nucleus was found after treatment [94]. Memantine was investigated in an open-label pilot trial in four HD patients and no differences in cortical or striatal metabolism were found between before and after treatment [95].

### Conclusion

There are no treatments currently available to stop disease progression in HD. Brain metabolism (relative bilateral increases in thalamic, occipital and cerebellar glucose metabolism associated with bilateral decreases in striatal [^18^F]FDG binding), postsynaptic dopaminergic function (reduction in striatal and cortical D_1_ receptor [^11^C]SCH22390 binding and D_2_ receptor [^11^C]raclopride binding) and PDE10A levels (reduction in striatal and pallidal [^11^C]IMA107 binding and increase in motor thalamic nuclei [^11^C]IMA107 binding) have been shown to be powerful biomarkers for assessing progression in patients with manifest HD and also during premanifest stages. However, no single biomarker may currently be considered optimal due to specific limitations of each ligand. An integrated multimodal imaging approach, combining different techniques, should be developed for evaluating potential neuroprotective and preventive treatments in HD.
